# Was Kraepelin Prescient Regarding Excitation-Inhibition Imbalance in Schizophrenia?

**DOI:** 10.1093/schizbullopen/sgag032

**Published:** 2026-08-05

**Authors:** Bernd Hinney, Pascal Missonnier, Peter Gass, Dragos Inta

**Affiliations:** Translational Psychiatry, Department of Community Health, University of Fribourg, Chemin du Musée 4, 1700 Fribourg, Switzerland; Translational Psychiatry, Department of Community Health, University of Fribourg, Chemin du Musée 4, 1700 Fribourg, Switzerland; Department of Psychiatry and Psychotherapy, Central Institute of Mental Health Mannheim, University of Heidelberg, J5, 68159 Mannheim, Germany; Translational Psychiatry, Department of Community Health, University of Fribourg, Chemin du Musée 4, 1700 Fribourg, Switzerland; Department of Biomedicine, University of Basel, Hebelstrasse 20, 4056 Basel, Switzerland; Food Research and Innovation Center (FRIC), University of Fribourg, Chemin du Musée 9, 1700 Fribourg, Switzerland

**Keywords:** NMDA receptor, gamma oscillations, parvalbumin-positive interneurons, mismatch negativity, D-serine, neurodevelopment, schizophrenia

## Abstract

Research on schizophrenia increasingly highlights altered excitation-inhibition regulation in cortical circuits. Excitatory Glutamatergic pyramidal cells propagate activity, whereas inhibitory Gamma Amino Butyric Acid (GABA) interneurons periodically inhibit this activity. Their interaction supports gamma synchronization and the integration of information, while dysfunctional glutamatergic transmission (especially at the N-methyl-D-aspartate receptor [NMDA]-receptor) disrupts information flow. This framework invites a restricted reconsideration of Kraepelin’s concept of dementia praecox. While Kraepelin erred in proposing a uniform, deteriorating course (schizophrenia is a heterogeneous neurodevelopmental spectrum disorder and many patients remain stable or recover) his emphasis on an early disturbance of mental integration in schizophrenia may have been prescient. In schizophrenia, cognitive differences often precede psychosis, while genetic, experimental, postmortem, electrophysiological, and pharmacological findings converge on aberrations in the ecosystem of the NMDA-receptor in schizophrenic patients and members of high-risk groups for schizophrenia. D-serine, glycine, kynurenic acid, and redox state are important co-regulators of the NMDA receptor and constitute possible therapeutic targets. Negative trials of D-serine and glycine-transporter inhibitors, however, challenge simple augmentation strategies while leaving the developmental circuit hypothesis open, which would call for much earlier interventions. We argue that the decisive test for this hypothesis are biomarker-defined studies near illness onset; precisely when gamma synchronization or mismatch negativity already indicates impaired coordination. Excitation-inhibition imbalance thus gives Kraepelin’s clinical observation a modern and experimentally tractable form.

Although modern research remains far from deciphering the causes of schizophrenia, an increasingly coherent picture suggests that cortical excitation-inhibition (E/I) balance is disturbed in the disorder. More precisely, evidence converges on the interaction between glutamatergic pyramidal cells and GABAergic interneurons. Pyramidal cells propagate activity through cortical networks, while interneurons determine which activity continues and for how long. When inhibition arrives too weakly or too late, competing neuronal assemblies overlap and the representation carried by each assembly becomes less precise. A disturbance can therefore arise even when the overall amount of excitation and inhibition appears unchanged, because the important variable is the rhythm of excitation and inhibition within the same processing interval.

The infrastructure underlying normal E/I balance is established during childhood and adolescence through processes of careful calibration, which condition cortical circuits to acquire the temporal organization required for adult cognition. During this period, parvalbumin-positive interneurons mature, inhibitory synapses strengthen, and long-range networks become increasingly able to synchronize their activity. Abnormalities in these processes can already be detected in young persons at clinical high risk for psychosis. The existence of these disturbances *before* a first schizophrenic episode thus makes E/I imbalance relevant to the developmental origin of schizophrenia itself.^1–6^

Therefore, on a phenomenological level, neurodegenerative disorders and schizophrenia share one conspicuous feature: both can impair the brain’s capacity to organize complex information into coherent thought and action.

Though Kraepelin did not know, and could not have known, the underlying circuit mechanisms, it is thus possible and instructive to reconsider his famous designation of schizophrenia as “*dementia praecox*” (= an early form of dementia). Kraepelin used the word dementia in a broader clinical sense than the term carries today. His concept referred to an early weakening of mental integration and to an unfavorable course, rather than to the plaques, tangles, and generalized neuronal loss that later defined the major neurodegenerative dementias.^7^^,^^8^ He grouped together syndromes whose symptoms differed because he believed that their course revealed a common underlying process.

Before developing this analogy, it is vital to identify the fundamental aspects in which Kraepelin erred. Schizophrenia lacks (on the microscopic level) the generalized neuronal loss, and (on the macroscopic level) the progressive loss of autonomy that characterize dementia.^9^ In fact, longitudinal studies that follow individuals, who go on to develop schizophrenia, describe early cognitive aberrations during childhood, further cognitive change around the first episode in some individuals, and relative constancy of symptoms after onset in many patients.^8^^,^^10^ This sequence is important because an early developmental limitation can produce a persistent cognitive gap without requiring continued neuronal destruction. A person may enter adolescence with slower acquisition of selected abilities, lose additional cognitive ground during the transition to psychosis, and then remain broadly stable for years. Also, schizophrenia’s clinical trajectories are highly variable. Outcome studies show substantial rates of remission and recovery after first-episode psychosis; pooled estimates are far more favorable compared to the inevitable deterioration occurring in dementia.^11^ While some people with schizophrenia experience persistent cognitive and functional disability, others regain their previous level of functioning or construct a stable and satisfying life despite residual symptoms.

From this, two important conclusions follow. First, schizophrenia is a neurodevelopmental spectrum disorder, not a dementia-like process. Second, its prognosis ranges from sustained recovery to persistent impairment and is in many cases considerably better than Kraepelin’s term implies. These conclusions set the important boundary of Kraepelin’sanalogy. They also make its remaining, productive element more visible: a disturbance of information processing.

Cognitive difficulties in schizophrenia extend far beyond performance on neuropsychological tests. They encompass deficits in volition and motivation and thus change broad categories of human intellect; at least in that sub-group of schizophrenic patients who develop so called “negative symptoms.” Schizophrenic patients with negative symptoms tend to lead a (relative to their premorbid level) reduced life in which they no longer aspire to follow their passions or interests, and they seem less apt as before to bring their life in accordance with their own values. Usually, patients with schizophrenia also have difficulties in generating flexible hypotheses with regard to the interpretation of their environment, which makes them prone to get stuck in rigid, fixed or even delusional thought patterns.

A productive way to reformulate all these seemingly disparate deficits in information-theory-based terminology is to see them as expressions of a loss of intellectual *context*. Context is what gives information its relevance.^12^^,^^13^ When context is weak, a thought is less effectively constrained by what came before it. Reasoning becomes less able to compare an immediate interpretation with an alternative. Also, an action may not be initiated if loss of context-awareness makes it harder for a person to realize how this specific performance may bring the overall goal nearer. This can contribute to reduced motivation and attenuated volition. Crucially, longitudinal studies place such cognitive differences years before the diagnostic threshold for schizophrenia is crossed.^4^^,^^10^ During this so-called “prodromal phase” (largely identical with the clinical high-risk state for schizophrenia), subtle failures of attention, learning, and processing are already apparent.^14^ These changes inhibit adequate contextualization and can make ordinary situations more difficult to interpret because the brain must rely more heavily on the most immediate or salient cue. The same limitation can weaken the correction of an initial interpretation when later information contradicts it and thus increase the risk for delusional thought patterns.^15^^,^^16^ Taken together, cognitive disturbances belong to the *formation* of the disorder, not merely to the aftermath of psychosis or its treatment.

Excitation-inhibition imbalance offers a mechanistic account of these deficits. A cortical representation (of a concept, a thought or a sentence) can only be operationalized by the brain if the involved neurons fire together and if competing activity is suppressed. The pyramidal-interneuron circuit performs this selection repeatedly, on a millisecond timescale. Disturbed inhibition shortens the period during which one representation dominates the network and permits unrelated activity to enter the same processing window. The result is a loss of contrast between signal and background. More excitation then produces less usable information, because the network activates broadly without selecting sharply. This apparent paradox explains why cortical disinhibition can coexist with cognitive slowing and impoverished behavior. Activity is present, but it no longer resolves efficiently into a stable representation that can guide the next mental operation. Within this model, altered glutamatergic transmission is pivotal because glutamate recruits both the pyramidal cells that carry a signal and the interneurons that delimit it. The initiating disturbance may differ across individuals, but the circuit consequence can converge on the same failure of temporal coordination.

What is striking about the relationship between E/I imbalance and disturbed glutamatergic transmission is that it integrates findings from otherwise separate areas of schizophrenia research. Genome-wide association studies place a substantial part of genetic risk in genes concerned with synaptic organization and glutamatergic signaling.^17^ Genetic risk factors for schizophrenia that involve excitatory neurotransmission prominently feature specific loci, such as those for the metabotropic glutamate receptor 3, the α-amino-3-hydroxy-5-methyl-4-isoxazolepropionic acid receptor (glutamate receptor 1), and subunit 2A of the N-methyl-D-aspartate receptor (NMDAR).^18^ Postmortem analysis of mRNA-expression patterns has confirmed these findings regarding the subunit 2A of the NMDAR.^19^ Rare coding variants with larger individual risks for schizophrenia point in the same direction.^20^

In the healthy cortex, gamma rhythm (the central rhythm of higher-order processing in the brain) arises through a recurrent feedback loop between glutamatergic pyramidal cells and GABAergic parvalbumin-positive, fast-spiking interneurons. Pyramidal firing recruits these interneurons, and the hyperpolarizing output of these interneurons then suppresses pyramidal firing for approximately one gamma cycle, after which the next assembly can become active. Repetition of this sequence produces a rhythm of roughly 30-80 Hz and gives successive neuronal assemblies distinct temporal positions.^3^^,^^4^ N-methyl-D-aspartate receptor signaling contributes to the maturation and effective recruitment of parvalbumin interneurons. This contribution is particularly important during development, when excitatory input helps determine which inhibitory connections are stabilized and how strongly they control pyramidal output. Experimental reduction of NMDAR function in cortical interneurons weakens inhibitory control, disinhibits pyramidal cells, and produces physiological and behavioral changes relevant to schizophrenia.^21–23^ The experimental effect also depends on timing: early postnatal disruption produces broader consequences than an equivalent manipulation after circuit maturation. This finding gives biological substance to the above-mentioned observation that cognitive abnormalities can precede the first psychotic episode by years. E/I imbalance in this setting leads to temporal dispersion: pyramidal neurons continue to fire, but their activity is less sharply confined to a common phase. Gamma synchronization weakens because the inhibitory signal no longer closes each processing interval with the required precision, which results in de-contextualization of the information.

This circuit account corresponds closely to electrophysiological findings in schizophrenia. Auditory stimulation evokes less reliable gamma-frequency synchronization in schizophrenic patients. Meta-analytic evidence confirms a substantial reduction of the 40-Hz auditory steady-state response in patients with schizophrenia.^24^^,^^25^ The same patient group also shows differences to controls in two important electroencephalogram (EEG) paradigms that test for evoked potentials: *P50 suppression* and *mismatch negativity*. When 2 acoustic signals are presented in rapid succession, healthy controls show a suppression of the P50 wave (a positive wave occurring approximately 50 ms post-stimulus) upon the second signal. This suppression is largely absent in schizophrenic patients. Patients with schizophrenia also show a robust reduction of mismatch negativity. Mismatch negativity is a central cognitive process that can be interpreted as an automatic deviancy recognition of the human brain and thus as a sign of healthy signal processing. Its expression is a negative EEG-wave 100-250 ms after the deviant stimulus. The reduction in mismatch negativity in schizophrenic patients is associated with poorer everyday functioning.^26^^,^^27^ Reduced mismatch negativity can also precede psychosis and predict transition or clinical outcome in high-risk samples.^15^^,^^16^

Further support for this cognitive model comes from NMDAR co-agonism. The NMDAR opens efficiently only when glutamate and a co-agonist—glycine or D-serine—occupy their respective binding sites (glycine and D-serine bind to the same site, commonly termed “glycine site”). D-serine is generated from L-serine by the enzyme serine racemase. Altered D-serine metabolism has been reported in cerebrospinal fluid and postmortem brain tissue in schizophrenia.^28^^,^^29^ Strikingly, gene variants coding for serine racemase have also been implicated in the development of schizophrenia.^28^ Co-agonist availability therefore provides a direct route by which metabolism can influence the pyramidal-interneuron circuit. D-serine and glycine can preferentially gate different receptor pools, which makes receptor location relevant to the effect of supplementation.^30^ NMDARs also differ in subunit composition, which alters channel kinetics and the duration of the synaptic signal.^19^^,^^30^ The biological effect of increasing a co-agonist thus depends on the specific receptor population present at that developmental stage. For example, kynurenic acid (an endogenous product of tryptophan metabolism) antagonizes the glycine site, reduces glutamatergic transmission and is *elevated* in cerebrospinal fluid and cortex in schizophrenia.^31–34^ Redox state provides a further control point: NMDAR possess a central regulatory site that is sensitive to whether the surrounding is in a chemically reduced or oxidized state. An oxidized state (i.e., the presence of free radicals or reactive oxygen species) attenuates the activity of the NMDAR while a chemically reduced state (effectuated for example by antioxidant systems like glutathione) increase the activity of the NMDAR. It has been shown that the levels of glutathione are reduced in schizophrenic patients.^35–37^

NMDAR throughput thus depends on a complex ecosystem of biochemical conditions at the synapse: a low D-serine concentration, an excess of kynurenic acid, or an oxidized receptor can each reduce the synaptic transmission that recruits inhibitory control. This also highlights how very divergent patterns may lead to the same disruption of E/I regulation.

Such a perspective also inspires another, more liberal way to read Kraepelin’s analogy: it implies an overlap between (normal) aging processes and schizophrenic processes. Of note, in the aged hippocampus, D-serine and serine-racemase expression decline together with NMDAR-dependent plasticity. Experimental D-serine supplementation can restore part of the impaired synaptic response.^38^^,^^39^ Age-related oxidation can suppress the same receptor and weaken long-term potentiation.^40^ Schizophrenia and physiological cognitive aging can therefore converge on reduced NMDAR-dependent plasticity through different developmental routes. In aging, the change follows decades of established circuit function. In schizophrenia, the relevant disturbance may develop while those circuits are still maturing.

Challenges for the NMDA-hypofunction model, however, come from studies that employed proton magnetic resonance spectroscopy to measure the level of glutamate and glutamine (or the sum of both) in different brain regions. These studies generally show a *heightened* level of glutamatergic metabolites in most brain regions, contrary to what the NMDA-hypofunction model would suggest.^41^ While the authors of the corresponding meta-analysis took these results to mean that glutamatergic excitotoxicity may play a role in schizophrenia, others have defended the hypofunction model and have suggested that heightened levels of glutamatergic metabolites may be a compensatory response of the brain to malfunctioning NMDAR.^42^

In turn, clinical trials have so far provided some, but no universal validation of the NMDA-hypofunction model of schizophrenia. A target-engagement study found dose-dependent enhancement of auditory learning and mismatch negativity with D-serine in patients with schizophrenia, and intensive cognitive training was associated with rises in serum D-serine that correlated with cognitive gains.^43^^,^^44^ Broader clinical efficacy has, however, been inconsistent. Low-dose D-serine failed to improve negative or cognitive symptoms in a multicenter trial with schizophrenic patients.^45^ The glycine-transporter inhibitor bitopertin failed to ameliorate negative symptoms in phase 3 studies, and the CONNEX program found no significant cognitive or functional benefit of iclepertin (another glycine-transporter inhibitor).^46^^,^^47^

These results bring the developmental timing of the hypothesis to the foreground. If altered NMDAR-dependent maturation shapes inhibitory circuits years *before* psychosis, increasing co-agonist availability during fully established disease likely comes too late. This proposition supports biomarker-defined studies with serine-supplementation in clinical-high-risk groups for schizophrenia (and thus in individuals who are in the *prodromal* phase of schizophrenia). Previous studies with this design were too short to come to meaningful conclusions as to whether D-serine-supplementation can reduce conversion risk to psychosis.^48^ Also, supplementation studies with the pro-drug L-Serine have so far not been undertaken in clinical-high-risk groups. In addition, it is very possible that supplementation would have to occur already years *before* the prodromal phase itself, given the long trajectory of neurodevelopment. However, it is so far unknown in what way this could be operationalized, as current markers of elevated schizophrenia risk (before onset of the prodromal phase) are too unreliable to allow for a meaningful formation of study-populations.

Finally, NMDAR antagonists (like ketamine) provide additional evidence for the paramount role of glutamatergic transmission in psychosis. In healthy volunteers, subanesthetic ketamine (but also its S[+] enantiomer esketamine) can produce positive symptoms, negative symptoms, and a characteristic disruption of cognition.^49^ Ketamine reduces context-dependent processing and mismatch negativity, and it disturbs working-memory-related prefrontal activation and connectivity.^50^^,^^51^ In one experimental study, enhancing glycine-site signaling reduced the detrimental psychotomimetic effects of ketamine.^52^ Pharmaceutical NMDAR blockade is therefore sufficient to transiently reproduce the full spectrum of the schizophrenic syndrome, and enhancement of NMDAR co-agonism reverses these symptoms. While schizophrenia develops over a much longer period, the ketamine model (in essence, a concrete manifestation of the NMDA-hypofunction model) may therefore showcase—in a time-lapsed fashion—the effects of pharmaceutically-induced E/I imbalance.

In sum, while no claim is made that the mechanisms described above can explain the totality of schizophrenic symptoms, Kraepelin may have been prescient in placing an early disturbance of mental integration into the center of schizophrenia. E/I imbalance gives this clinical observation a modern, testable form. It links developmental risk to the pyramidal-interneuron circuit, this circuit to gamma synchronization, and gamma synchronization to the organization of information. This 3-step model also explains why cognitive abnormalities can precede psychosis and why interventions applied late in the illness may fail despite valid molecular targets. The model also converts the prodromal period from a vague prelude into a phase in which a measurable circuit operation may already be changing. Its central prediction is straightforward: early cognitive change should coincide with impaired NMDAR-dependent coordination of cortical excitation and inhibition. The next generation of studies should test whether restoring this coordination during the period in which symptoms are emerging changes the subsequent course of illness. In this restricted but consequential sense, Kraepelin’s historical analogy points toward a distinctly modern question: whether schizophrenia begins when the developing cortex loses the precision required to turn neuronal activity into coherent and contextualized thought.

## Data Availability

No new data were generated for this article.
